# Quality of life and anorectal function in rectal cancer patients undergoing immunotherapy-based total neoadjuvant therapy: a comparison of organ preservation and radical surgery

**DOI:** 10.1016/j.ctro.2026.101275

**Published:** 2026-08-29

**Authors:** Luoxi He, Zonglin Lv, Qianyu Zhou, Ruone Xu, Kewen Hu, Siyuan Chen, Yingxuan Lin, Shuwen Li, Yujun Liu, Mingxu Yan, Lili Huang, Weiqing Lu, Zhiyuan Zhang, Yajie Chen, Hui Zhang, Menglong Zhou, Wang Yang, Juefeng Wan, Ruiyan Wu, Lijun Shen, Shujuan Zhou, Yan Wang, Jingwen Wang, Ye Xu, Xinxiang Li, Sanjun Cai, Zhen Zhang, Fan Xia, Yaqi Wang

**Affiliations:** a Department of Radiation Oncology, Fudan University Shanghai Cancer Center, Shanghai, 200032, China; b Department of Oncology, Shanghai Medical College, Fudan University, Shanghai, 200032, China; c Shanghai Clinical Research Center for Radiation Oncology, Shanghai, 200032, China; d Shanghai Key Laboratory of Radiation Oncology, Shanghai, 200032, China; e Cancer Institute, Fudan University Shanghai Cancer Center, Shanghai, 200032, China; f Department of Colorectal Surgery, Fudan University Shanghai Cancer Center, Shanghai, 200032, China

**Keywords:** Quality of life, Anorectal function, Total neoadjuvant therapy, Immunotherapy, Organ preservation, Rectal cancer

## Abstract

**Purpose:**

Immunotherapy-based total neoadjuvant therapy (iTNT) improves clinical complete response rates and increases organ preservation (OP) opportunities in rectal cancer, while its long-term impacts on quality of life (QoL) and anorectal function remain unclear. This study aimed to evaluate long-term QoL and anorectal function after iTNT, and to clarify how OP versus radical surgery affects patient-reported outcomes and functional recovery, providing evidence for individualized clinical decision-making.

**Material and methods:**

This prospective longitudinal cohort study enrolled rectal cancer patients receiving iTNT from 2021 to 2024. Patient-reported outcomes were dynamically evaluated using the EORTC QLQ-C30/CR29, Low Anterior Resection Syndrome (LARS), Wexner, and Memorial Sloan Kettering Cancer Center Bowel Function Instrument (MSKCC-BFI) questionnaires. Longitudinal changes were analyzed using linear mixed-effects models accounting for within-patient correlation. A separate conventional TNT cohort was included for exploratory cross-sectional comparison of long-term outcomes. Risk factors for major LARS were evaluated using univariable and multivariable logistic regression with multiple imputation and complete-case sensitivity analyses.

**Results:**

Of 172 patients receiving iTNT, 142 were eligible for QoL analysis and 137 for anorectal function assessment after excluding five patients with permanent stomas. Patients managed with OP were associated with better global health status, lower symptom burden, and better anorectal function than those undergoing total mesorectal excision (TME), with lower LARS and Wexner scores during follow-up. Compared with conventional TNT, poorer QoL and anorectal outcomes after iTNT were mainly observed in patients undergoing TME, whereas outcomes were largely comparable among those managed with OP. Multivariate analysis identified TME and shorter tumor distance from the anal verge as independent risk factors for major LARS.

**Conclusion:**

OP following iTNT was associated with more favorable long-term QoL and anorectal functional outcomes than TME. These findings support OP strategies as an important approach for optimizing long-term patient-reported outcomes in appropriately selected patients with rectal cancer.

## Highlights

• Organ-preserving strategies provide superior long-term QoL and anorectal function compared with radical TME.
• iTNT-associated long-term functional impairment is mainly observed in patients undergoing TME.
• Long-term functional outcomes after OP remain comparable between iTNT and conventional TNT.
• Shorter tumor distance from the anal verge and TME are independent risk factors for major LARS.

## 1 Introduction

Organ preservation (OP) has emerged as a major therapeutic goal in rectal cancer treatment, including both low-lying early rectal cancer and locally advanced rectal cancer (LARC) [1]. Although total mesorectal excision (TME) remains the standard treatment for many patients [2], radical surgery is frequently associated with substantial morbidity, including bowel dysfunction and persistent deterioration in patient-reported QoL [3]. Consequently, organ-preserving approaches such as watch-and-wait (W&W) or local excision (LE) following a favorable neoadjuvant response have gained increasing acceptance, offering the opportunity to avoid radical resection while maintaining QoL and anorectal function in selected patients [1], [4], [5].

Successful OP largely depends on achieving a complete or near-complete tumor response before definitive treatment [6]. Total neoadjuvant therapy (TNT), which integrates neoadjuvant chemoradiotherapy (nCRT) with systemic chemotherapy delivered before surgery, has been demonstrated to improve tumor response, reduce distant metastasis and increase the possibility of OP [7], [8], [9]. More recently, the incorporation of immunotherapy into TNT (iTNT) has demonstrated promising clinical efficacy even in proficient mismatch repair (pMMR) or microsatellite stable (MSS) tumors [10], [11], [12], [13], [14]. These regimens significantly increase the rate of clinical complete response (cCR) and expand eligibility for organ-preserving strategies such as W&W [10], [12], [13], [15].

However, improved tumor response does not always equate to better patient-reported outcomes. Pelvic radiotherapy is known to impair bowel function and may contribute to chronic defecatory symptoms after treatment [16]. In addition, immunotherapy may also induce immune-related adverse events (irAEs), including colitis and mucosal inflammation [17], which may further exacerbate gastrointestinal toxicity when combined with TNT. In the setting of nCRT plus immunotherapy for LARC, the reported incidence of grade 3–4 diarrhea was 2%, and grade 3–4 immune-related adverse events occurred in 4% (range 2–5%) [18], with immune-related colitis occurring in 0.9–2% of patients in UNION, TORCH and SPRING-01 trials [10], [11], [13]. Severe gastrointestinal toxicity may also delay or terminate treatment: in our TORCH trial which performed iTNT approach, all 9 patients with grade 3–4 gastrointestinal adverse events experienced treatment delays exceeding 1 month, and 2 patients with severe immune-related colitis permanently discontinued chemoimmunotherapy [10]. Importantly, while OP aims to preserve anorectal function and avoid the morbidity associated with TME, evidence regarding the impact of iTNT-treated OP strategies on long-term QoL and anorectal function in rectal cancer remains limited.

Therefore, this study aims to evaluate long-term QoL and anorectal function in patients with rectal cancer who were managed with OP or TME following iTNT, addressing the current paucity of evidence on patient-reported outcomes in this emerging treatment paradigm.

## 2 Material and methods

### 2.1 Patient cohorts

This study was approved by the Ethics Committee of Fudan University Shanghai Cancer Center (FUSCC). From May 2021 to March 2024, a cohort of non-metastatic pMMR/MSS rectal cancer patients who were treated with the iTNT strategy at FUSCC was included in this study. This cohort comprised patients enrolled in the prospective clinical trials TORCH (NCT04518280, LARC, May 2021–September 2022) and TORCH-E (NCT05555888, low-lying early rectal cancer, December 2022–March 2024), as well as patients receiving the same regimens. Longitudinal questionnaire data were collected at three time points: before treatment (baseline), after treatment, and at the most recent long-term follow-up, enabling prospective assessment of trajectories in QoL and anorectal function over the course of iTNT treatment.

A total of 172 patients were initially enrolled in the iTNT cohort. Patients were excluded according to the following criteria: death during follow-up (*n* = 4), local disease progression (*n* = 10), and distant metastasis (*n* = 16). These patients were excluded because disease progression or metastatic disease could substantially affect QoL and functional outcomes independent of the effects of neoadjuvant treatment and OP strategies. After these exclusions, 142 eligible patients remained for subsequent analyses and were included in the QoL assessment.

For anorectal functional analyses, an additional 5 patients with permanent stomas were excluded because anorectal function cannot be meaningfully evaluated in the absence of an intact anorectal pathway. These patients remained included in the QoL analysis, as several domains of the EORTC QLQ-C30 and QLQ-CR29 remain applicable to patients with stomas. Therefore, 137 patients were included in the anorectal function analysis.

Patients who were unreachable by telephone or submitted incomplete questionnaires were excluded from corresponding longitudinal questionnaire analyses. Valid questionnaires were collected at three predefined time points: before treatment, after completion of iTNT, and during long-term follow-up. For QoL evaluation, the number of evaluable patients at each time point was 92 (64.7%), 97 (68.3%) and 91 (64.1%), respectively. For anorectal function assessment, evaluable cases at each time point were 86 (62.8%), 83 (60.6%) and 103 (75.2%), respectively. All participants provided written informed consent. Patient characteristics are summarized in Table 1.

**Table 1:** Patient characteristics, treatments and efficacy evaluation.

Characteristics	QoL				Anorectal function			
	Total (*n* = 142)	OP (*n* = 57)	TME (*n* = 85)	*P*	Total (*n* = 137)	OP (n = 57)	TME (*n* = 80)	*P*
Age, years, Median (Q1,Q3)	55 (48, 63)	55 (48, 64)	55 (47, 62)	0.734	55 (48, 64)	53 (48, 64)	55.5 (47.75, 63.25)	1
Sex, n (%)				0.705				0.774
Female	96 (67.61)	37 (64.91)	59 (69.41)		92 (67.15)	37 (64.91)	55 (68.75)	
Male	46 (32.39)	20 (35.09)	26 (30.59)		45 (32.85)	20 (35.09)	25 (31.25)	
Tumor distance from anus, cm, Median (Q1,Q3)	3.5 (2.1, 5)	3 (2, 4)	4 (3, 6)	0.002	3.5 (2.1, 5)	3 (2, 4)	4 (3, 5.93)	< 0.001
TNM stage, n (%)				0.003				0.003
I	8 (5.63)	6 (10.53)	2 (2.35)		7 (5.11)	5 (8.77)	2 (2.5)	
II	22 (15.49)	14 (24.56)	8 (9.41)		22 (16.06)	15 (26.32)	7 (8.75)	
III	112 (78.87)	37 (64.91)	75 (88.24)		108 (78.83)	37 (64.91)	71 (88.75)	
cT, n (%)				< 0.001				< 0.001
2	14 (9.86)	11 (19.3)	3 (3.53)		15 (10.95)	11 (19.3)	4 (5)	
3	109 (76.76)	46 (80.7)	63 (74.12)		106 (77.37)	45 (78.95)	61 (76.25)	
4	19 (13.38)	0 (0)	19 (22.35)		16 (11.68)	1 (1.75)	15 (18.75)	
cN, n (%)				< 0.001				< 0.001
0	31 (21.83)	21 (36.84)	10 (11.76)		30 (21.9)	21 (36.84)	9 (11.25)	
1	48 (33.8)	26 (45.61)	22 (25.88)		51 (37.23)	26 (45.61)	25 (31.25)	
2	63 (44.37)	10 (17.54)	53 (62.35)		56 (40.88)	10 (17.54)	46 (57.5)	
cMRF, n (%)				0.006				0.016
Negative	103 (72.54)	49 (85.96)	54 (63.53)		102 (74.45)	49 (85.96)	53 (66.25)	
Positive	39 (27.46)	8 (14.04)	31 (36.47)		35 (25.55)	8 (14.04)	27 (33.75)	
cEMVI, n (%)				0.002				0.001
Negative	113 (79.58)	53 (92.98)	60 (70.59)		108 (78.83)	53 (92.98)	55 (68.75)	
Positive	29 (20.42)	4 (7.02)	25 (29.41)		29 (21.17)	4 (7.02)	25 (31.25)	
ycT, n (%)				< 0.001				< 0.001
0	44 (30.99)	31 (54.39)	13 (15.29)		44 (32.12)	31 (54.39)	13 (16.25)	
1	3 (2.11)	1 (1.75)	2 (2.35)		4 (2.92)	1 (1.75)	3 (3.75)	
2	43 (30.28)	21 (36.84)	22 (25.88)		40 (29.2)	20 (35.09)	20 (25)	
3	42 (29.58)	4 (7.02)	38 (44.71)		40 (29.2)	4 (7.02)	36 (45)	
4	10 (7.04)	0 (0)	10 (11.76)		9 (6.57)	1 (1.75)	8 (10)	
ycN, n (%)				< 0.001				0.007
0	115 (80.99)	54 (94.74)	61 (71.76)		112 (81.75)	53 (92.98)	59 (73.75)	
1	22 (15.49)	2 (3.51)	20 (23.53)		21 (15.33)	3 (5.26)	18 (22.5)	
2	5 (3.52)	1 (1.75)	4 (4.71)		4 (2.92)	1 (1.75)	3 (3.75)	
ycMRF, n (%)				0.028				0.045
0	130 (91.55)	56 (98.25)	74 (87.06)		127 (92.7)	56 (98.25)	71 (88.75)	
1	12 (8.45)	1 (1.75)	11 (12.94)		10 (7.3)	1 (1.75)	9 (11.25)	
ycEMVI, n (%)				0.003				0.021
0	128 (90.14)	57 (100)	71 (83.53)		124 (90.51)	56 (98.25)	68 (85)	
1	14 (9.86)	0 (0)	14 (16.47)		13 (9.49)	1 (1.75)	12 (15)	
Induction chemotherapy, n (%)				1				0.815
No	98 (69.01)	39 (68.42)	59 (69.41)		91 (66.42)	39 (68.42)	52 (65)	
Yes	44 (30.99)	18 (31.58)	26 (30.59)		46 (33.58)	18 (31.58)	28 (35)	
CR, n (%)				< 0.001				< 0.001
No	63 (44.37)	4 (7.02)	59 (69.41)		58 (42.34)	5 (8.77)	53 (66.25)	
Yes	79 (55.63)	53 (92.98)	26 (30.59)		79 (57.66)	52 (91.23)	27 (33.75)	
Decision after iTNT/TNT, n (%)				< 0.001				< 0.001
OP	57 (40.14)	57 (100)	0 (0)		57 (41.61)	57 (100)	0 (0)	
TME	85 (59.86)	0 (0)	85 (100)		80 (58.39)	0 (0)	80 (100)	

**NOTE.** Data are No. (%) unless otherwise indicated. The number of patients differs between quality of life (QoL) and anorectal function analyses due to the availability of questionnaire data. Patients with colostomy were excluded from anorectal function analyses.

**Abbreviations:** cEMVI, clinical extramural vascular invasion; cMRF, clinical mesorectal fascia involvement; cN, clinical nodal stage; cT, clinical tumor stage; CR, complete response; iTNT, total neoadjuvant therapy combined with immunotherapy; Q1, first quartile; Q3, third quartile; QoL, quality of life; TME, total mesorectal excision; TNT, total neoadjuvant therapy; OP,organ preservation; ycEMVI, post-treatment extramural vascular invasion; ycMRF, post-treatment mesorectal fascia involvement; ycN, post-treatment nodal stage; ycT, post-treatment tumor stage.

A separate cohort of patients treated with conventional TNT was included for cross-sectional comparison of long-term QoL and anorectal function. Because of the different cohort design, only long-term follow-up data were available for this comparison.

### 2.2 Treatments and efficacy evaluation

Patients in the iTNT cohort received short-course radiotherapy (SCRT, 25Gy/5 fractions) followed by 4–6 cycles of CAPOX combined with PD-1 inhibitor. Patients in the TNT cohort received long-course chemoradiotherapy (LCRT, 50Gy/25 fractions concurrent with oral capecitabine, with or without irinotecan), followed by at least 4 cycles of consolidation chemotherapy. After neoadjuvant therapy, tumor restaging was evaluated by digital rectal examination (DRE), endoscopy, rectal MRI and thoracic and abdominal CT. Patients achieving cCR were offered an option of W&W strategy, while others underwent local excision (LE) or TME. Patients who developed tumor regrowth during W&W were suggested to undergo salvage TME. CR was defined as either sustained cCR in patients managed with W&W or pathological CR (pCR) confirmed by surgical histopathology. OP was defined as maintaining rectal integrity, including the adoption of W&W and LE.

### 2.3 Assessment of QoL and anorectal function

Quality of life and anorectal function were assessed by trained research staff through structured telephone interviews using validated questionnaires. General QoL was assessed by the European Organization for Research and Treatment of Cancer-Quality of Life Questionnaire-C30 (EORTC QLQ-C30) and colorectal cancer specific QoL was assessed with the EORTC QLQ-CR29 [19], [20]. Anorectal functional outcomes were measured via the Low Anterior Resection Syndrome (LARS) score, Wexner incontinence and Memorial Sloan Kettering Cancer Center Bowel Function Instrument (MSKCC-BFI) questionnaire [21], [22], [23]. These questionnaires were not administered to patients with a colostomy.

### 2.4 Statistical analysis

Longitudinal changes in QoL and anorectal function scores across time and treatment groups were assessed using linear mixed-effects models (LMMs). Fixed effects included treatment strategy, time, and the treatment-by-time interaction term, while patient ID was fitted as a random intercept to account for repeated measurements within individuals. To further reduce potential confounding, we used multivariable LMMs adjusted for clinically relevant baseline characteristics, including age, sex, tumor distance from the anal verge, clinical T stage (cT), clinical N stage (cN), clinical mesorectal fascia involvement (cMRF), and clinical extramural vascular invasion (cEMVI). Raw *P*-values for multiple outcome indicators were adjusted for multiple testing using the Benjamini–Hochberg false discovery rate (FDR) method.

Missing data patterns for baseline and post-treatment variables were summarized. Among the 191 patients included in risk factor analyses for major LARS, missing values were observed for clinical staging variables: cT (*n* = 2 missing), cN (*n* = 3 missing), ycT stage, ycN stage, post-treatment mesorectal fascia involvement (ycMRF) status and post-treatment extramural vascular invasion (ycEMVI) status (*n* = 23 missing for each variable). Multiple imputation by chained equations (MICE) was implemented as the primary strategy to handle missing covariate data. Values were drawn with predictive mean matching. The number of imputations was set to 10. Complete-case analysis was performed as a sensitivity analysis to test the robustness of regression findings.

To explore potential factors for major LARS (score ≥ 30), we performed univariate association of 16 candidate variables, including demographics (age, sex), treatment parameters (induction chemotherapy, the addition of immunotherapy, surgery), tumor characteristics (tumor distance from anal verge, stage, cT, cN, cMRF, cEMVI, ycT, ycN, ycMRF, ycEMVI), and CR status. Variables with *P* < .15 in univariable analysis were entered into a multivariable logistic regression model.

Prior to multivariable logistic regression model, collinearity between predictors was assessed using variance inflation factor (VIF), with substantial collinearity (VIF > 10) were excluded. Events per variable (EPV) was calculated to verify adequate statistical power for multivariate logistic regression. Hosmer–Lemeshow test was used for goodness-of-fit. Results are reported as odds ratios (ORs) with 95% confidence intervals, and *P* < .05 (two-sided) was considered statistically significant.

## 3 Results

### 3.1 Patient characteristics

A total of 142 eligible patients were included in the study. Five patients with permanent stomas were excluded from the anorectal function analysis because validated anorectal function questionnaires are not applicable in patients without an intact anorectal passage. Baseline characteristics of the QoL cohort (*n* = 142) and the anorectal function cohort (*n* = 137) are summarized in Table 1.

As expected in this non-randomized cohort, patients managed with OP differed significantly from those undergoing TME. Compared with the TME group, patients in the OP group had lower clinical stage, lower cT stage, lower nodal burden, less MRF involvement, less EMVI, and substantially higher CR rates.

**Fig. 1:**
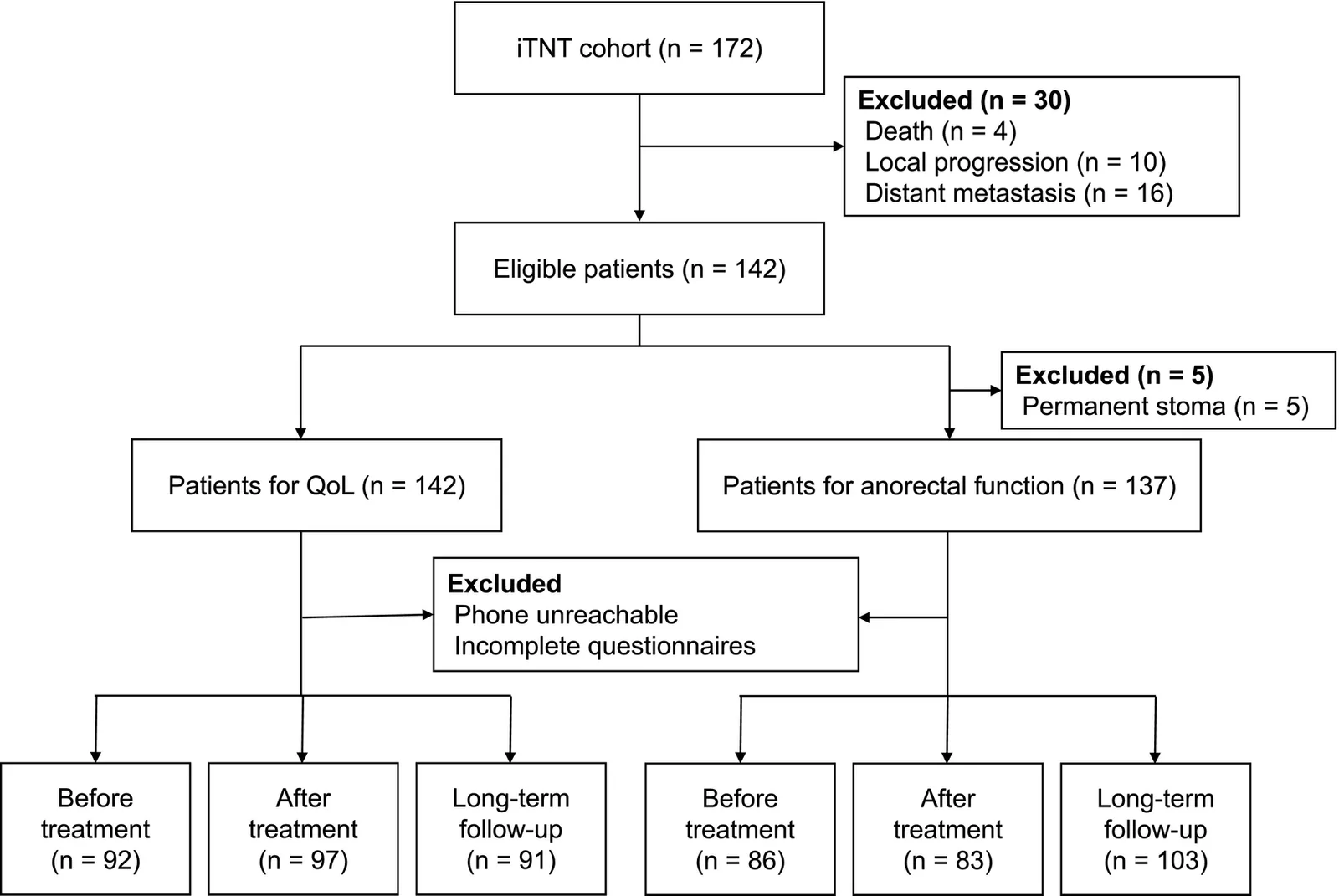
Workflow diagram of patient selection.

Patients who were unreachable by telephone or returned incomplete questionnaires were excluded from the corresponding analyses at each assessment time point. For the QoL analysis, questionnaires were available for 92 patients (64.7%) at baseline, 97 (68.3%) after treatment, and 91 (64.1%) at long-term follow-up. For the anorectal function analysis, the corresponding numbers were 86 (62.8%), 83 (60.6%), and 103 (75.2%), respectively (Fig. 1).

To evaluate the potential for selection bias related to questionnaire completion, baseline characteristics were compared among patients with available questionnaires at baseline, post-treatment, and long-term follow-up. No significant differences were observed across the total and three assessment time points, suggesting that questionnaire completion was not associated with baseline clinical characteristics (Tables S1).

### 3.2 Temporal Dynamics of QoL and Anorectal Function Comparing OP and TME in Patients Receiving iTNT

**Fig. 2:**
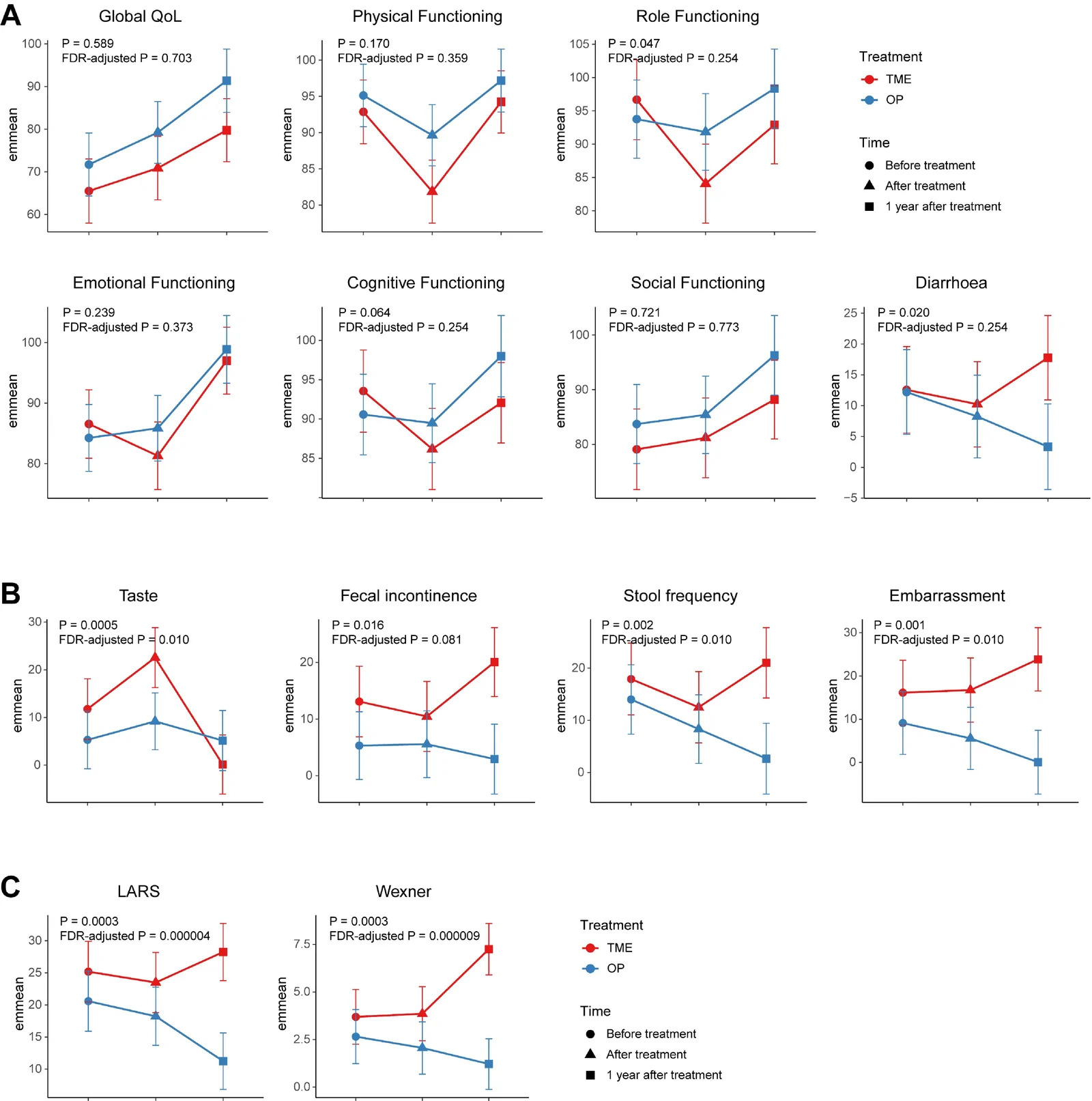
QoL and anorectal function from baseline to long-term follow-up comparing OP and TME in iTNT. (A) Selected EORTC QLQ-C30 outcomes of iTNT patients in baseline, after treatment and long-term follow-up comparing OP and TME. Longitudinal trajectories were estimated using LMMs with time, treatment group, and their interaction as fixed effects and patient-specific random intercepts. Data are presented as estimated marginal means (EMMs) with 95% confidence intervals. *P* values for the time-by-treatment interaction were adjusted for multiple comparisons using the Benjamini–Hochberg FDR procedure. (B) Selected EORTC QLQ-CR29 outcomes of iTNT patients in baseline, after treatment and long-term follow-up comparing OP and TME. Longitudinal trajectories were estimated using LMMs with time, treatment group, and their interaction as fixed effects and patient-specific random intercepts. Data are presented as EMMs with 95% confidence intervals. *P* values for the time-by-treatment interaction were adjusted for multiple comparisons using the Benjamini–Hochberg FDR procedure. (C) Anorectal function of iTNT patients measured by LARS (left) and Wexner (right) score in baseline, after treatment and long-term follow-up comparing OP and TME. Longitudinal trajectories were estimated using LMMs with time, treatment group, and their interaction as fixed effects and patient-specific random intercepts. Data are presented as EMMs with 95% confidence intervals. *P* values for the time-by-treatment interaction were adjusted for multiple comparisons using the Benjamini–Hochberg FDR procedure.

LMMs demonstrated significant time effects for most QoL domains, reflecting overall recovery following treatment. Improvements over time were observed in several domains, including global QoL, social functioning, emotional functioning, pain, and constipation in the QLQ-C30, as well as urinary frequency, abdominal pain, buttock pain, blood and mucus in stool, dry mouth, bloating, anxiety, and body image in the QLQ-CR29. Following treatment, both strategies experienced transient deterioration in role functioning, cognitive functioning, fatigue, nausea/vomiting, and appetite loss on the QLQ-C30, together with taste disturbance and sore skin on the QLQ-CR29. These impairments subsequently recovered during long-term follow-up, suggesting that they primarily reflected reversible treatment-related toxicities (Fig. 2A, B**;** Fig. S1, S2**;** Table S2).

Significant treatment effects were observed for several domains, including global QoL, physical functioning, emotional functioning, and social functioning, indicating that patients managed with OP consistently reported better outcomes than those undergoing TME across the study period. In contrast, significant treatment-by-time interactions were identified for taste disturbance, stool frequency, and embarrassment (all FDR-adjusted *P* < .05), indicating that the trajectories of these symptoms differed significantly between the OP and TME treatments over time (Fig. 2A, B**;** Fig. S1, S2**;** Table S2).

Longitudinal anorectal functional assessments further supported these findings. Significant treatment-by-time interactions were observed for both LARS and Wexner scores (both FDR-adjusted *P* < .05), indicating distinct recovery trajectories between treatment strategies (Fig. 2C**;** Table S2). Specifically, LARS scores remained largely unchanged, while Wexner incontinence scores worsened in the TME group throughout long-term follow-up. In contrast, patients managed with OP demonstrated progressive improvement in both LARS and Wexner scores over time, suggesting superior preservation and recovery of anorectal function.

### 3.3 Comparative Analysis of QoL and Anorectal Function Between iTNT and Conventional TNT Cohorts

To provide additional context regarding longer-term patient-reported outcomes, we included a separate cohort of patients treated with conventional TNT with extended follow-up. As iTNT represents a relatively recent treatment strategy, the follow-up duration of the iTNT cohort was shorter than that of the conventional TNT cohort. The median follow-up duration was 21 months (IQR, 18–27.5 months) for QoL assessment and 21 months (IQR, 16–27 months) for anorectal function assessment in the iTNT cohort, compared with 41 months (IQR, 25–59 months) and 48 months (IQR, 33–62 months), respectively, in the conventional TNT cohort. Given the differences in treatment regimens, including radiotherapy approaches, systemic therapy exposure, immunotherapy administration, and follow-up duration, comparisons between these cohorts should be interpreted cautiously.

**Fig. 3:**
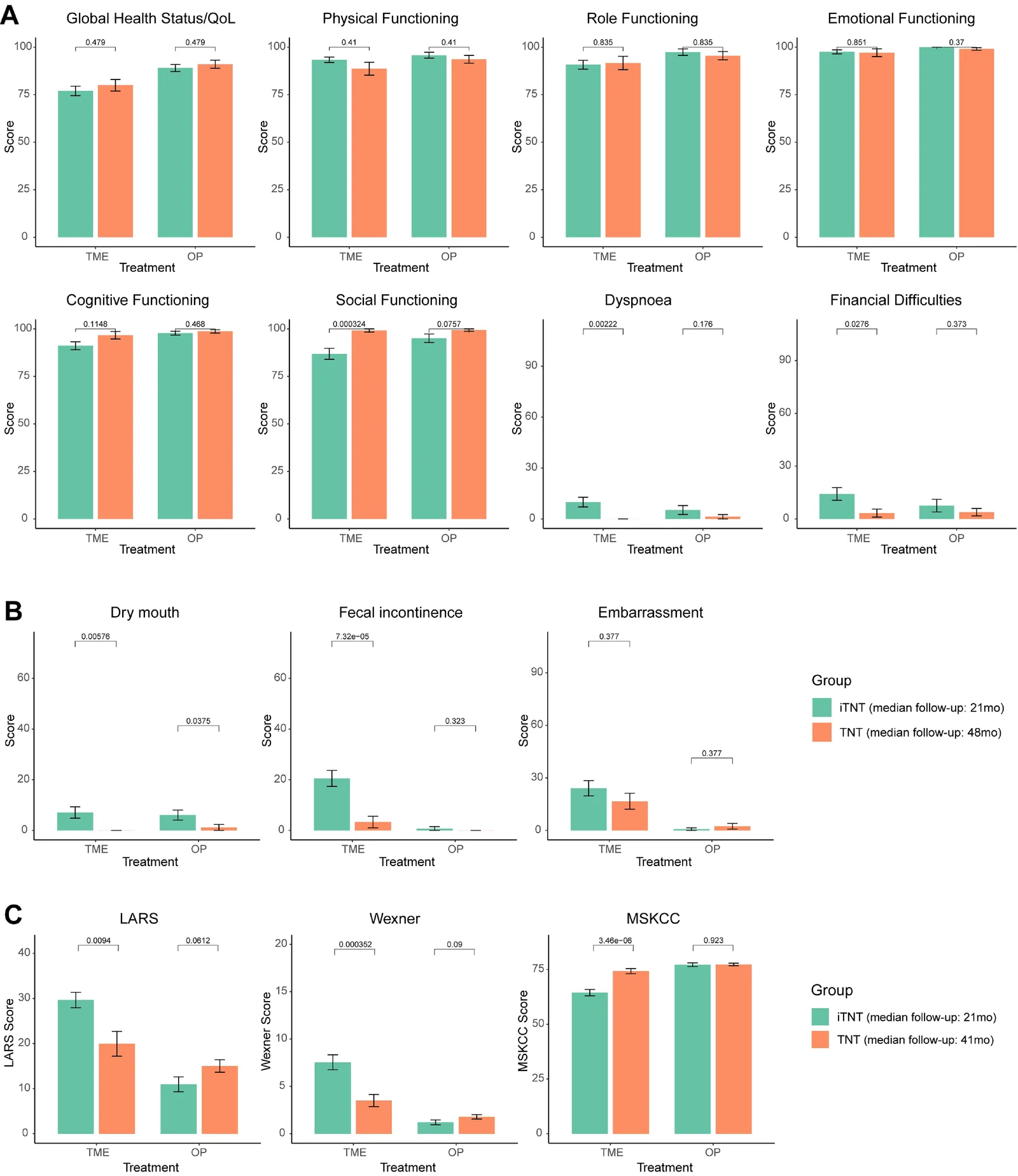
Long-term QoL and anorectal function comparing OP with TME after iTNT and TNT. (A) Selected EORTC QLQ-C30 outcomes during long-term follow-up between iTNT and TNT patients comparing OP and TME. Median follow-up was 21 months (IQR, 18–27.5 months) for the iTNT cohort and 41 months (IQR, 25–59 months) for the conventional TNT cohort. Between-group comparisons were performed using Student's *t-*tests. *P* values were adjusted for multiple comparisons using the Benjamini–Hochberg FDR procedure. (B) Selected EORTC QLQ-CR29 outcomes during long-term follow-up between iTNT and TNT patients comparing OP and TME. Median follow-up was 21 months (IQR, 18–27.5 months) for the iTNT cohort and 41 months (IQR, 25–59 months) for the conventional TNT cohort. Between-group comparisons were performed using Student's *t-*tests. *P* values were adjusted for multiple comparisons using the Benjamini–Hochberg FDR procedure. (C) Anorectal function measured by LARS (left), Wexner (middle) and MSKCC-BFI (right) score during long-term follow-up between iTNT and TNT patients comparing OP and TME. Median follow-up was 21 months (IQR, 16–27 months) for the iTNT cohort and 48 months (IQR, 33–62 months) for the conventional TNT cohort. Between-group comparisons were performed using independent-samples *t-*tests, with *P* values adjusted for multiple comparisons using the Benjamini–Hochberg FDR procedure.

Overall, QoL outcomes were broadly comparable between patients receiving iTNT and conventional TNT. Several domains demonstrated differences between the two cohorts, particularly among patients undergoing TME. In the TME subgroup, patients treated with iTNT reported worse social functioning and greater symptom burden, including dyspnea and financial difficulties on the EORTC QLQ-C30. Similarly, higher symptom scores for dry mouth and fecal incontinence on the EORTC QLQ-CR29 were observed in the iTNT cohort. In contrast, among patients managed with OP, most QoL domains were comparable between the iTNT and TNT cohorts despite differences in treatment composition (Fig. 3A, B**;** Fig. S3, S4).

Consistent with QoL findings, differences in anorectal function between iTNT and conventional TNT were mainly observed among patients undergoing TME. Compared with the iTNT cohort, patients receiving conventional TNT had better anorectal functional scores, including lower LARS and Wexner scores and higher MSKCC-BFI scores. Conversely, no significant differences in anorectal function were observed among patients managed with OP (Fig. 3C).

Collectively, these findings suggest that patients achieving OP after iTNT had comparable long-term QoL and anorectal functional outcomes to those treated with conventional TNT. However, the observed differences among patients undergoing TME should be interpreted in the context of differences in treatment intensity, treatment era, and follow-up duration between cohorts. Further studies with longer follow-up and more comparable treatment backgrounds are required to determine the long-term functional impact of immunotherapy-based TNT.

### 3.4 Risk Factors Associated with Major LARS Following Treatment

To identify potential predictors of poor anorectal function, patients were categorized according to their LARS scores into major LARS (≥ 30) and minor/no LARS (< 30). Univariate analysis identified several variables associated with major LARS (*P* < .15), including lower tumor distance from the anal verge, higher cT stage (cT3–4), presence of cEMVI, ycT3–4 stage, the addition of immunotherapy, CR status, and TME. These variables were subsequently entered into the multivariable logistic regression model. No evidence of multicollinearity was observed among the candidate variables, and the model included seven variables with 56 major LARS events (EPV = 8). The model demonstrated good calibration according to the Hosmer–Lemeshow goodness-of-fit test (*P* = .823).

Multivariable analysis identified lower tumor distance from the anal verge (OR = 0.67, 95% CI = 0.53–0.81, *P* < .001) and TME (OR = 13.63, 95% CI = 3.69–50.33, *P* < .001) as independent predictors of major LARS (Table 2). Complete-case sensitivity analyses yielded consistent results, supporting the robustness of the findings (Table S3).

**Table 2:** Univariate and Multivariate Analyses of Risk Factors for Major LARS.

Characteristic		No/Minor LARS (*N* = 135)	Major LARS (*N* = 56)	OR (univariable)	OR (multivariable)
Age, years	Mean ± SD	56.4 ± 10.7	55.8 ± 10.1	0.99 (0.97–1.02, *p* = .714)	
Sex	Male	91 (67.4%)	41 (73.2%)		
	Female	44 (32.6%)	15 (26.8%)	0.76 (0.38–1.51, *p* = .430)	
Tumor distance from anus, cm	Mean ± SD	4.0 ± 2.3	3.5 ± 1.6	0.88 (0.76–1.03, *p* = .121)	0.65 (0.53–0.81, *p* < .001)
cT	1–2	37 (27.4%)	7 (12.5%)		
	3–4	98 (72.6%)	49 (87.5%)	2.64 (1.10–6.36, *p* = .030)	1.16 (0.41–3.29, *p* = .776)
cN	Negative	39 (28.9%)	14 (25%)		
	Positive	96 (71.1%)	42 (75%)	1.22 (0.60–2.48, *p* = .585)	
cMRF	Negative	112 (83%)	44 (78.6%)		
	Positive	23 (17%)	12 (21.4%)	1.33 (0.61–2.90, *p* = .476)	
cEMVI	Negative	114 (84.4%)	39 (69.6%)		
	Positive	21 (15.6%)	17 (30.4%)	2.37 (1.13–4.94, *p* = .022)	1.31 (0.53–3.24, *p* = .561)
ycT	1–2	58 (43%)	16 (28.6%)		
	3–4	77 (57%)	40 (71.4%)	1.88 (0.96–3.69, *p* = .065)	1.60 (0.70–3.70, *p* = .268)
ycN	Negative	124 (91.9%)	48 (85.7%)		
	Positive	11 (8.1%)	8 (14.3%)	1.88 (0.71–4.95, *p* = .203)	
ycMRF	Negative	131 (97%)	54 (96.4%)		
	Positive	4 (3%)	2 (3.6%)	1.21 (0.22–6.82, *p* = .827)	
ycEMVI	Negative	128 (94.8%)	52 (92.9%)		
	Positive	7 (5.2%)	4 (7.1%)	1.41 (0.39–5.01, *p* = .599)	
Addition of immunotherapy	No	62 (45.9%)	36 (64.3%)		
	Yes	73 (54.1%)	20 (35.7%)	2.13 (1.11–4.00, *p* = .022)	0.84 (0.53–1.19, *p* = .671)
Induction chemotherapy	No	114 (84.4%)	44 (78.6%)		
	Yes	21 (15.6%)	12 (21.4%)	1.48 (0.67–3.26, *p* = .330)	
CR	No	26 (19.3%)	29 (51.8%)		
	Yes	109 (80.7%)	27 (48.2%)	0.22 (0.11–0.44, *p* < .001)	1.08 (0.31–3.77, *p* = .908)
Decision after iTNT/TNT	OP	102 (75.6%)	18 (32.1%)		
	TME	33 (24.4%)	38 (67.9%)	6.53 (3.29–12.94, p < .001)	13.63 (3.69–50.33, p < .001)

**Note.** Continuous variables are presented as mean ± standard deviation (SD). Variables with *P* < .15 in univariable analysis were included in the multivariable model.

**Abbreviations:** CR, complete response; LARS, low anterior resection syndrome; LE, local excision; OR, odds ratio; TME, total mesorectal excision; TNT, total neoadjuvant therapy; OP, organ preservation; cEMVI, clinical extramural vascular invasion; cMRF, clinical mesorectal fascia involvement; cN, clinical nodal stage; cT, clinical tumor stage; iTNT, total neoadjuvant therapy combined with immunotherapy; ycEMVI, post-treatment extramural vascular invasion; ycMRF, post-treatment mesorectal fascia involvement; ycN, post-treatment nodal stage; ycT, post-treatment tumor stage.

To further evaluate the influence of treatment strategy on anorectal outcomes, subgroup analyses were performed in patients undergoing TME and those managed with OP. The OP cohort included both W&W and LE approaches, with 114 patients managed by W&W and 6 patients undergoing LE. Given the limited number of LE patients, separate analyses of W&W and LE were not performed.

In the TME subgroup, lower tumor distance from the anal verge (OR = 0.52, 95% CI = 0.37–0.73, *P* < .001) and the addition of immunotherapy (OR = 3.84, 95% CI = 1.11–12.50, *P* = .033) were independently associated with major LARS (Table S4). However, because patients receiving iTNT had a substantially shorter follow-up than those receiving conventional TNT, this association should be interpreted with caution. In contrast, no significant predictors were identified in the OP subgroup, likely reflecting the overall preservation of anorectal function in patients managed without radical surgery (Table S5).

## 4 Discussion

In this study, we demonstrated that OP following iTNT was associated with long-term advantages in patient-reported QoL and anorectal function compared with TME. Although both treatment pathways exhibited dynamic recovery trajectories over time, patients managed with OP consistently were associated with superior outcomes across multiple functional and symptom domains, together with significantly lower LARS and Wexner scores during long-term follow-up. Importantly, comparative analyses between TNT and iTNT cohorts further suggested that long-term QoL and anorectal functional outcomes among patients managed with OP were broadly comparable between treatment strategies, despite differences in immunotherapy exposure, radiotherapy approaches, systemic treatment regimens, and follow-up duration.

Several QoL domains improved over time in both the OP and TME groups, which likely reflects the benefits of tumor control, symptom relief, and sustained treatment response after completion of combined immunotherapy [24], [25]. Improvements in cancer-related symptoms, including pain, constipation, abdominal discomfort, and psychosocial distress, may substantially contribute to this recovery process. In contrast, the transient deterioration observed shortly after treatment in domains such as fatigue, appetite loss, cognitive and role functioning, and treatment-related local symptoms was most likely attributable to the acute toxicities of chemoradiotherapy and immunotherapy [26]. The subsequent recovery of these measures further supports the notion that many early impairments are reversible and predominantly treatment-related rather than permanent.

The divergence between treatment strategies was particularly pronounced in anorectal functional outcomes. Patients treated with TME demonstrated limited improvement in LARS over time, whereas Wexner incontinence scores remained persistently impaired during follow-up [27]. By contrast, patients managed with OP showed continuous improvement in both LARS and Wexner scores throughout follow-up. Consistent with previous studies, these findings further support the long-term functional advantages of W&W strategies [3]. This observation is clinically important, as bowel dysfunction remains one of the leading causes of long-term dissatisfaction after rectal cancer treatment despite technically successful surgery. Most patients in our cohort had low rectal cancer, a subgroup that bears the highest burden of functional impairment following radical pelvic surgery. Moreover, our risk-factor analysis identified TME and shorter tumor distance from the anal verge as independent predictors of major LARS, further emphasizing the functional consequences of radical pelvic surgery. Collectively, these findings indicate that successful OP was associated with more favorable long-term anorectal functional outcomes, a benefit that may be especially meaningful for patients with low-lying tumors.

The comparison between TNT and iTNT cohorts provides additional context regarding patient-reported outcomes after intensified neoadjuvant therapy. Among patients undergoing TME, those treated with iTNT showed less favorable outcomes in several QoL domains and anorectal function compared with patients receiving conventional TNT. However, these findings should be interpreted cautiously given the differences in follow-up duration, radiotherapy strategies, systemic treatment exposure, and treatment era between the two cohorts. In contrast, among patients managed with OP, QoL and anorectal functional outcomes were largely comparable between iTNT and TNT cohorts despite differences in treatment composition. These observations suggest that the favorable functional outcomes associated with OP may persist across different treatment approaches, although prospective studies with longer follow-up are required to confirm this finding.

Importantly, recent studies have demonstrated that incorporating immune checkpoint blockade into neoadjuvant treatment may improve CR rates in LARC, including increased pCR and cCR rates compared with conventional chemoradiotherapy [18], [28]. These findings provide a rationale for further investigating whether iTNT may facilitate OP strategies in appropriately selected patients. From the perspective of patient-reported outcomes, these findings raise the possibility that the functional advantages observed in patients managed with OP may be related to the avoidance of radical surgery, although this hypothesis requires prospective validation.

This study has several limitations. First, as a non-randomized observational study, treatment allocation was influenced by clinical characteristics and treatment response, resulting in potential selection bias between OP and TME groups. Although multivariable adjustment was performed, residual confounding cannot be completely excluded. Second, the comparison between iTNT and conventional TNT cohorts is limited by marked differences in follow-up duration and heterogeneity in radiotherapy strategy, systemic therapy, immunotherapy exposure, and treatment era, which preclude definitive conclusions regarding comparative recovery trajectories. Third, patients with local progression, distant metastases, or missing follow-up data may have had worse outcomes, and their exclusion from some analyses could lead to overestimation of functional recovery. Fourth, the sample size remains limited, particularly for subgroup analyses and risk-factor modeling. The relatively small number of major LARS events may have reduced the precision of multivariable estimates, as reflected by the relatively wide confidence intervals of some ORs. Future studies with larger cohorts and longer follow-up are warranted to validate these findings.

## 5 Conclusion

In conclusion, OP following iTNT was associated with more favorable long-term QoL and anorectal functional outcomes compared with TME. Although iTNT may improve CR rates and expand opportunities for OP, whether enhanced tumor response directly translates into improved functional outcomes requires further prospective validation. The favorable patient-reported outcomes observed after OP may be partly related to avoidance of radical surgery. For appropriately selected patients with rectal cancer, OP strategies offer a valuable approach to preserving long-term QoL and anorectal function.

## Ethics approval and consent to participate

Our study was approved by Ethics Committee of FUSCC (No. 2009224–7) and was conducted in accordance with the Declaration of Helsinki and informed consent was obtained from all participants.

## Availability of data and materials

The data utilized in this study can be obtained from the corresponding author upon reasonable request.

## CRediT authorship contribution statement

**Luoxi He:** Methodology, Investigation, Validation, Data curation, Visualization, Writing – original draft. **Zonglin Lv:** Methodology, Investigation, Validation, Visualization, Writing – original draft. **Qianyu Zhou:** Methodology, Investigation, Validation, Visualization, Writing – review & editing. **Ruone Xu:** Methodology. **Kewen Hu:** Methodology. **Siyuan Chen:** Resources. **Yingxuan Lin:** Resources. **Shuwen Li:** Resources. **Yujun Liu:** Resources. **Mingxu Yan:** Resources. **Lili Huang:** Resources. **Weiqing Lu:** Resources. **Zhiyuan Zhang:** Resources. **Yajie Chen:** Resources. **Hui Zhang:** Resources. **Menglong Zhou:** Resources. **Wang Yang:** Resources. **Juefeng Wan:** Resources. **Ruiyan Wu:** Resources. **Lijun Shen:** Resources. **Shujuan Zhou:** Resources. **Yan Wang:** Resources. **Jingwen Wang:** Resources. **Ye Xu:** Resources. **Xinxiang Li:** Resources. **Sanjun Cai:** Resources. **Zhen Zhang:** Conceptualization, Resources, Supervision, Project administration, Funding acquisition. **Fan Xia:** Conceptualization, Methodology, Resources, Supervision, Project administration, Writing – review & editing. **Yaqi Wang:** Conceptualization, Methodology, Resources, Supervision, Project administration, Writing – review & editing.

## Consent for publication

Not applicable.

## Ethics declaration

### Informed consent and patient details

Written informed consent to take part in the study and to publish the article has been obtained from all participants or their legal representatives. The privacy rights of participants have been observed.

### Studies in Human

This study was performed in compliance with relevant laws, regulatory frameworks and guidelines where the research took place.

This study was approved by the Ethics Committee of Fudan University Shanghai Cancer Center.

(Approval No. 2009224–7)

## Funding

This study was supported by the 10.13039/501100001809National Natural Science Foundation of China No. 82272732, 10.13039/501100001809National Natural Science Foundation of China No. 82473237, and Shanghai Hospital Development Center Three-Year Action Plan for Clinical Research (No. SHDC2020CR3082B).

## Declaration of competing interest

The authors declare that they have no known competing financial interests or personal relationships that could have appeared to influence the work reported in this paper.

## Appendix A Supplementary Data

Supplementary material 1 **Figure S1. EORTC QLQ-C30 outcomes of iTNT patients in baseline, after treatment and long-term follow-up comparing OP and TME****.** Longitudinal trajectories were estimated using LMMs with time, treatment group, and their interaction as fixed effects and patient-specific random intercepts. Data are presented as estimated marginal means (EMMs) with 95% confidence intervals. P values for the time-by-treatment interaction were adjusted for multiple comparisons using the Benjamini–Hochberg false discovery rate (FDR) procedure (Table S2).

Supplementary material 2 **Figure S2. EORTC QLQ-CR29 outcomes of iTNT patients in baseline, after treatment and long-term follow-up comparing OP and TME****.** Longitudinal trajectories were estimated using LMMs with time, treatment group, and their interaction as fixed effects and patient-specific random intercepts. Data are presented as EMMs with 95% confidence intervals. P values for the time-by-treatment interaction were adjusted for multiple comparisons using the Benjamini–Hochberg FDR procedure (Table S2).

Supplementary material 3 **Figure S3. EORTC QLQ-C30 outcomes during long-term follow-up between iTNT and TNT patients comparing OP and TME. Median follow-up: iTNT 21 mo; TNT 48 mo.** Median follow-up was 21 months (IQR, 18–27.5 months) for the iTNT cohort and 41 months (IQR, 25–59 months) for the conventional TNT cohort. Between-group comparisons were performed using Student’s *t* tests. P values were adjusted for multiple comparisons using the Benjamini–Hochberg FDR procedure.

Supplementary material 4 **Figure S4. EORTC QLQ-CR29 outcomes during long-term follow-up between iTNT and TNT patients comparing OP and TME. Median follow-up: iTNT 21 mo; TNT 48 mo.** Median follow-up was 21 months (IQR, 18–27.5 months) for the iTNT cohort and 41 months (IQR, 25–59 months) for the conventional TNT cohort. Between-group comparisons were performed using Student’s *t* tests. P values were adjusted for multiple comparisons using the Benjamini–Hochberg FDR procedure.

Supplementary material 5 Table S1. Baseline characteristics of patients with available questionnaires at each follow-up time point

Supplementary material 6 Table S2. Unadjusted and FDR-adjusted P-values for comparisons of QoL and anorectal function across treatment and time points

Supplementary material 7 Table S3. Univariate and multivariate analyses of risk factors for major LARS using complete-case data

Supplementary material 8 Table S4. Univariate and multivariate analyses of risk factors for major LARS in TME subgroup

Supplementary material 9 Table S5. Univariate and multivariate analyses of risk factors for major LARS in OP subgroup
