# Supplementary material for: Quality of life and anorectal function in rectal cancer patients undergoing immunotherapy-based total neoadjuvant therapy: a comparison of organ preservation and radical surgery
Source: Clin Transl Radiat Oncol. 2026 Aug 29;61:101275. doi: 10.1016/j.ctro.2026.101275 (PMC13571426; doi:10.1016/j.ctro.2026.101275)
Supplement: Supplementary file 1 [file mmc1.pdf]

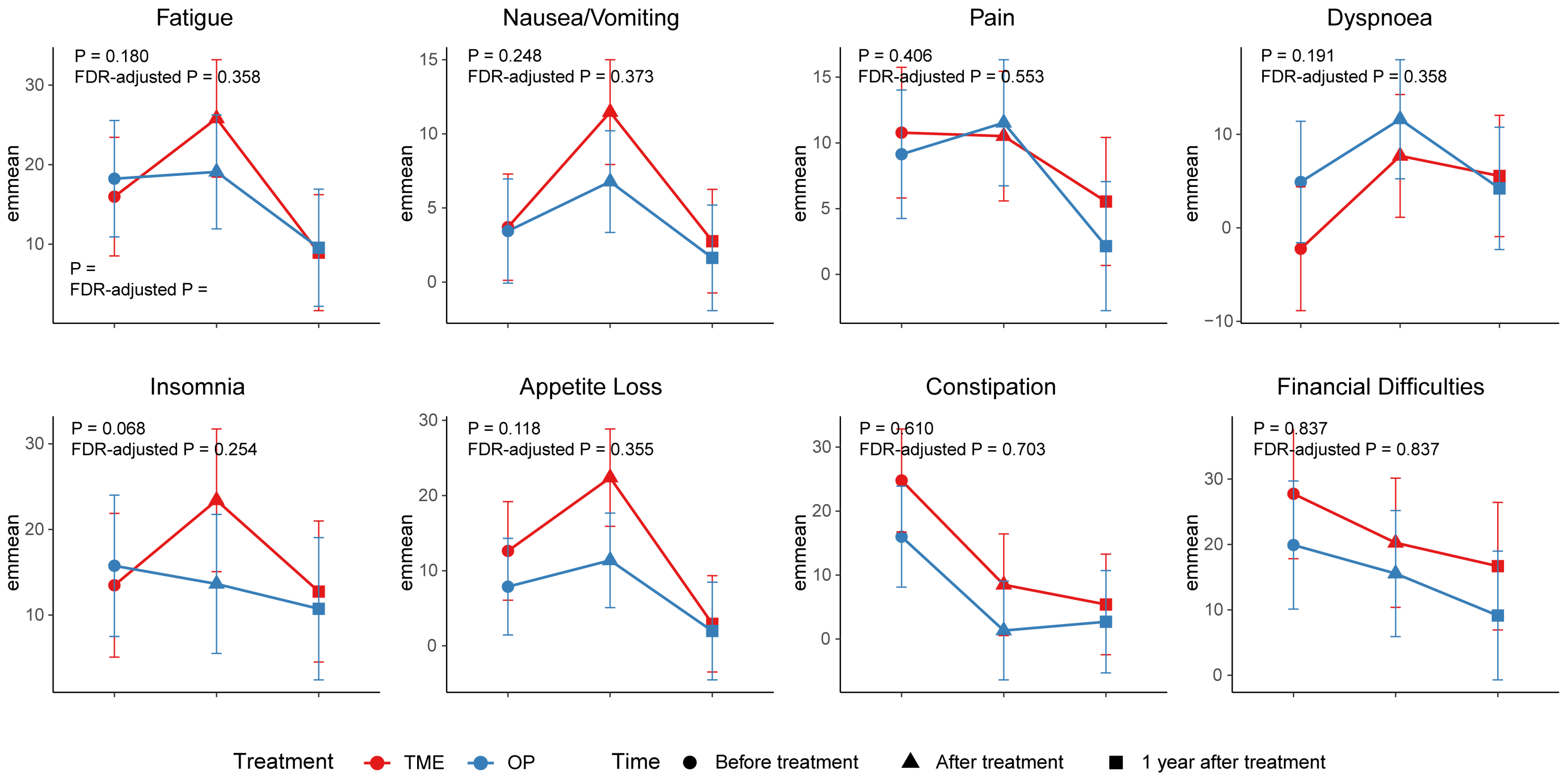

Fatigue
P = 0.180
FDR-adjusted P = 0.358
P =
FDR-adjusted P =
Nausea/Vomiting
P = 0.248
FDR-adjusted P = 0.373
Pain
P = 0.406
FDR-adjusted P = 0.553
Dyspnoea
P = 0.191
FDR-adjusted P = 0.358
Insomnia
P = 0.068
FDR-adjusted P = 0.254
Appetite Loss
P = 0.118
FDR-adjusted P = 0.355
Constipation
P = 0.610
FDR-adjusted P = 0.703
Financial Difficulties
P = 0.837
FDR-adjusted P = 0.837
emmean
Treatment
TME
OP
Time
Before treatment
After treatment
1 year after treatment
