# Supplementary material for: Quality of life and anorectal function in rectal cancer patients undergoing immunotherapy-based total neoadjuvant therapy: a comparison of organ preservation and radical surgery
Source: Clin Transl Radiat Oncol. 2026 Aug 29;61:101275. doi: 10.1016/j.ctro.2026.101275 (PMC13571426; doi:10.1016/j.ctro.2026.101275)
Supplement: Supplementary file 2 [file mmc2.pdf]

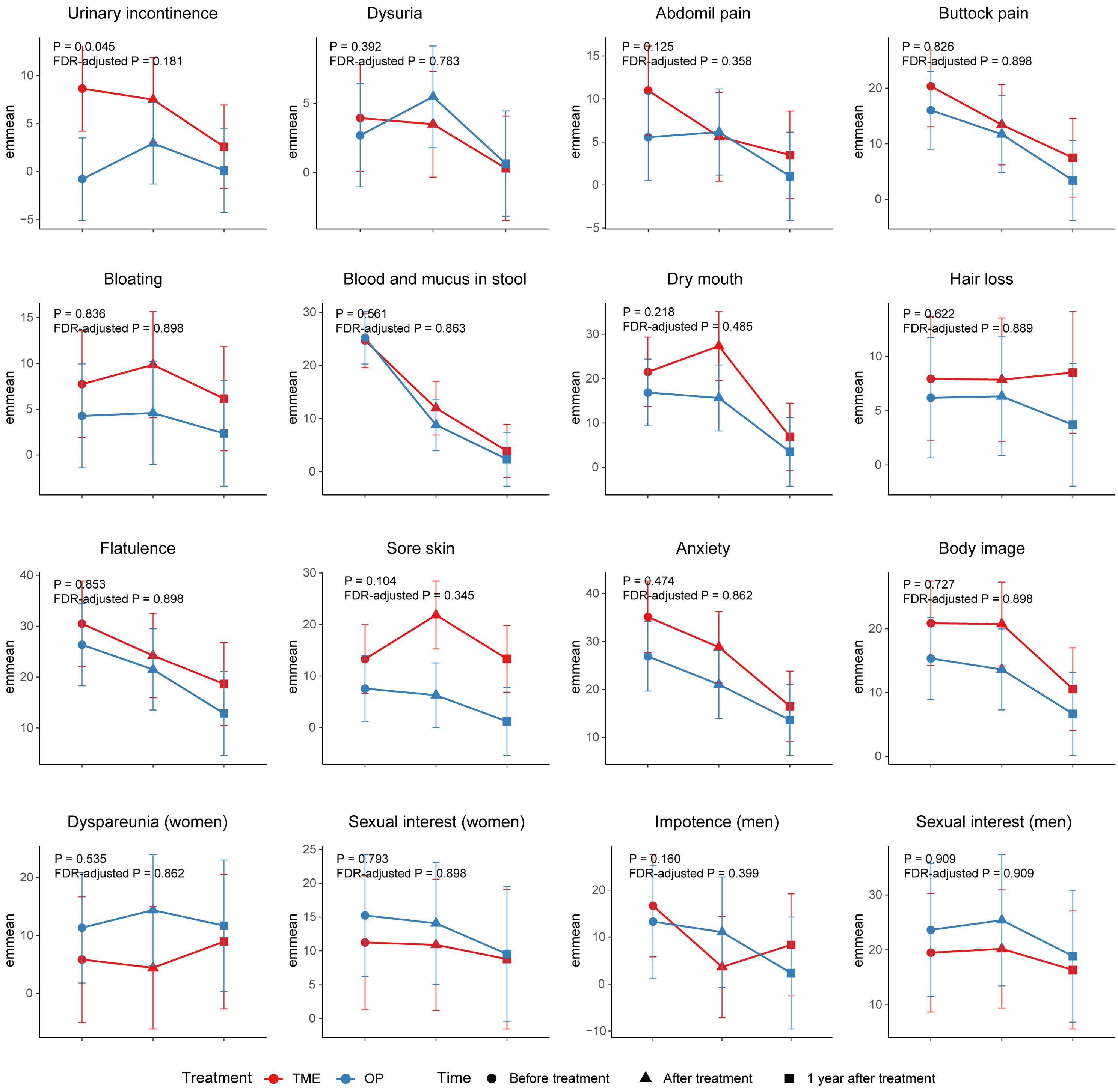

Urinary incontinence
P = 0.0.045
FDR-adjusted P = 0.181
Dysuria
P = 0.392
FDR-adjusted P = 0.783
Abdomil pain
P = 0.125
FDR-adjusted P = 0.358
Buttock pain
P = 0.826
FDR-adjusted P = 0.898
Bloating
P = 0.836
FDR-adjusted P = 0.898
Blood and mucus in stool
P = 0.561
FDR-adjusted P = 0.863
Dry mouth
P = 0.218
FDR-adjusted P = 0.485
Hair loss
P = 0.622
FDR-adjusted P = 0.889
Flatulence
P = 0.853
FDR-adjusted P = 0.898
Sore skin
P = 0.104
FDR-adjusted P = 0.345
Anxiety
P = 0.474
FDR-adjusted P = 0.862
Body image
P = 0.727
FDR-adjusted P = 0.898
Dyspareunia (women)
P = 0.535
FDR-adjusted P = 0.862
Sexual interest (women)
P = 0.793
FDR-adjusted P = 0.898
Impotence (men)
P = 0.160
FDR-adjusted P = 0.399
Sexual interest (men)
P = 0.909
FDR-adjusted P = 0.909
emmean
Treatment TME OP
Time Before treatment After treatment 1 year after treatment
