# Supplementary material for: Quality of life and anorectal function in rectal cancer patients undergoing immunotherapy-based total neoadjuvant therapy: a comparison of organ preservation and radical surgery
Source: Clin Transl Radiat Oncol. 2026 Aug 29;61:101275. doi: 10.1016/j.ctro.2026.101275 (PMC13571426; doi:10.1016/j.ctro.2026.101275)
Supplement: Supplementary file 3 [file mmc3.pdf]

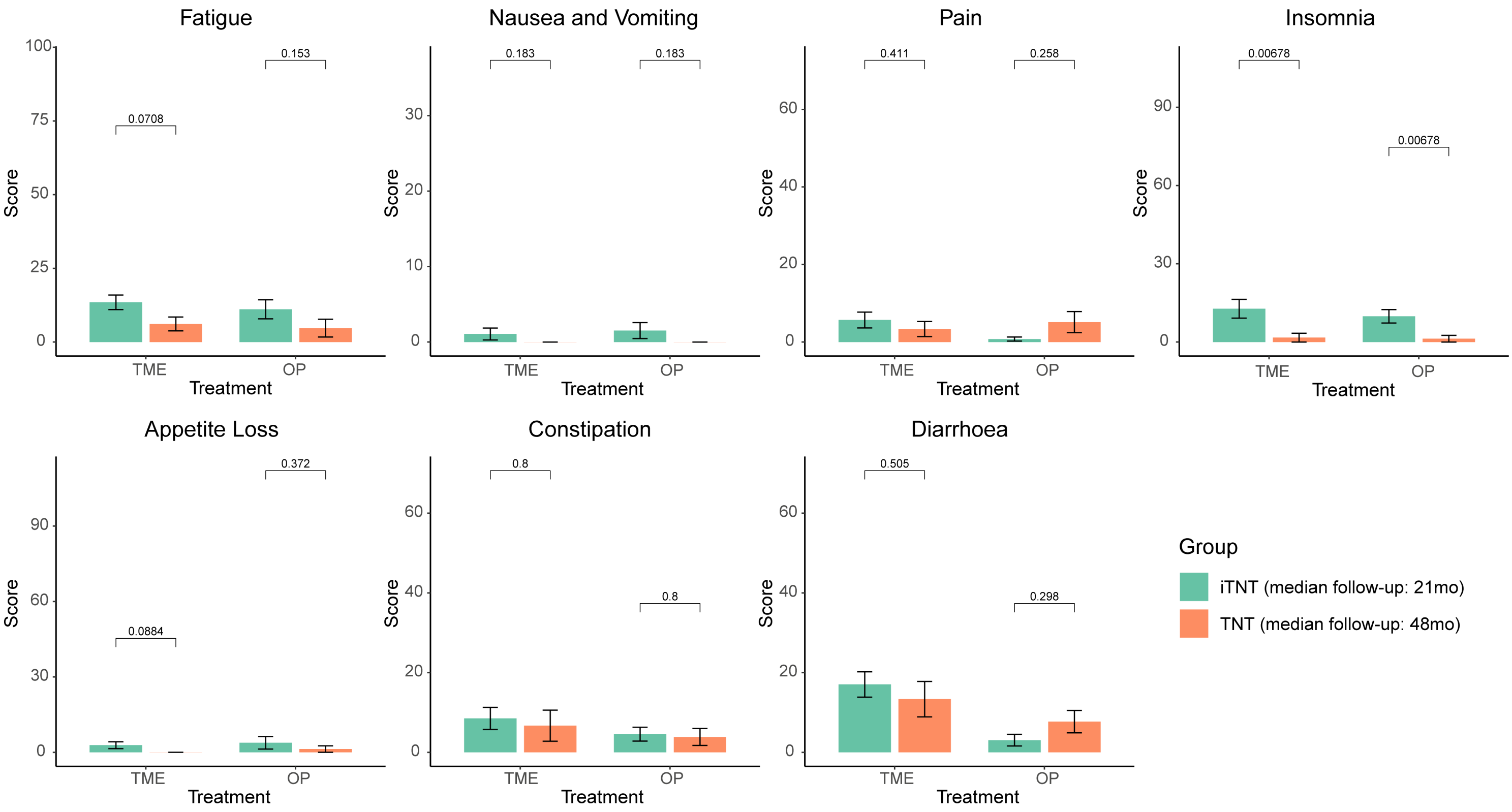

Fatigue
Nausea and Vomiting
Pain
Insomnia
Appetite Loss
Constipation
Diarrhoea
Score
Treatment
TME
OP
0.0708
0.153
0.183
0.183
0.411
0.258
0.00678
0.00678
0.0884
0.372
0.8
0.8
0.505
0.298
Group
iTNT (median follow-up: 21mo)
TNT (median follow-up: 48mo)
