# Supplementary material for: Quality of life and anorectal function in rectal cancer patients undergoing immunotherapy-based total neoadjuvant therapy: a comparison of organ preservation and radical surgery
Source: Clin Transl Radiat Oncol. 2026 Aug 29;61:101275. doi: 10.1016/j.ctro.2026.101275 (PMC13571426; doi:10.1016/j.ctro.2026.101275)
Supplement: Supplementary file 4 [file mmc4.pdf]

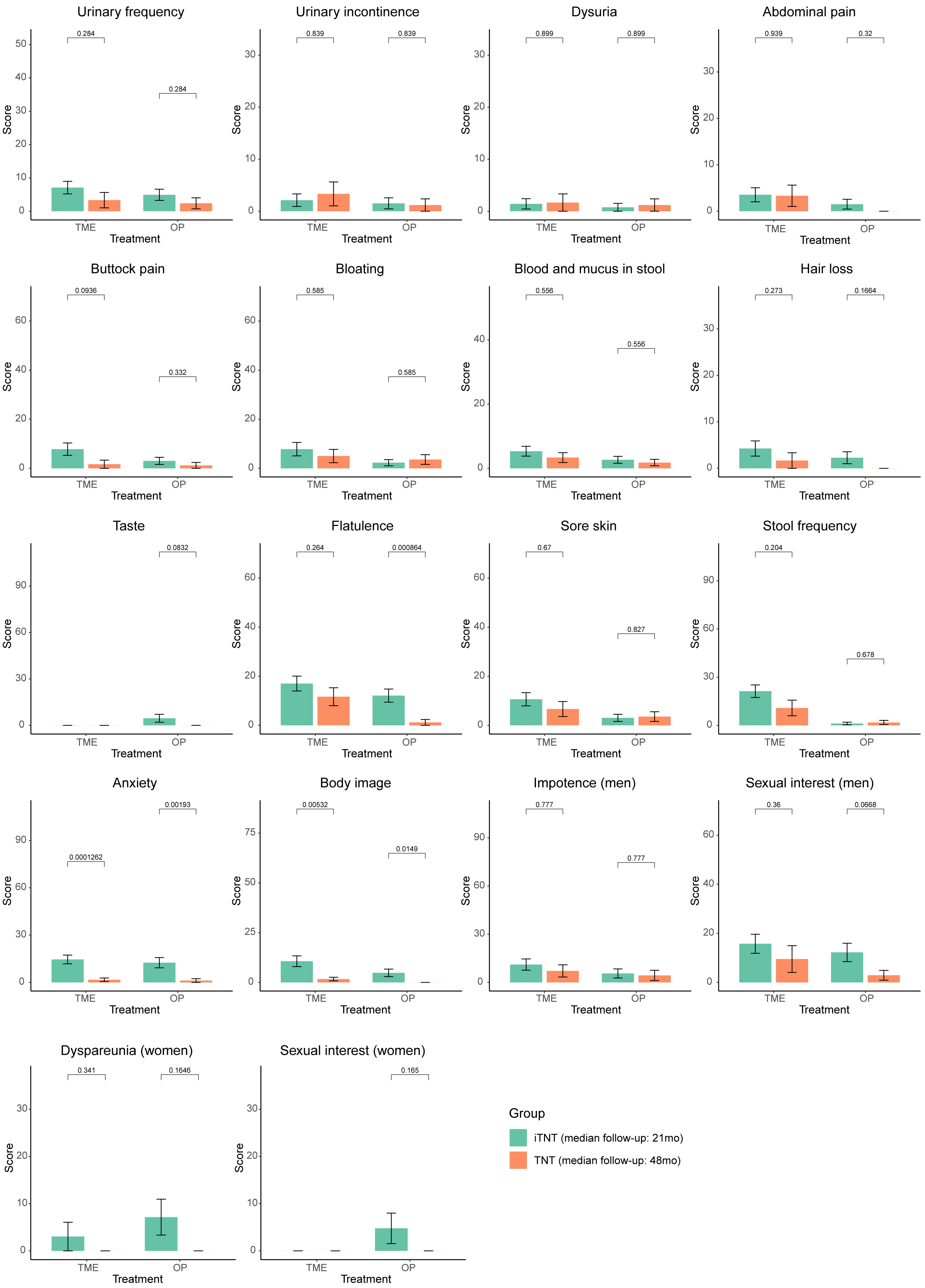

Urinary frequency
0.284
0.284
Urinary incontinence
0.839
0.839
Dysuria
0.899
0.899
Abdominal pain
0.939
0.32
Buttock pain
0.0936
0.332
Bloating
0.585
0.585
Blood and mucus in stool
0.556
0.556
Hair loss
0.273
0.1664
Taste
0.0832
Flatulence
0.264
0.000864
Sore skin
0.67
0.827
Stool frequency
0.204
0.678
Anxiety
0.0001262
0.00193
Body image
0.00532
0.0149
Impotence (men)
0.777
0.777
Sexual interest (men)
0.36
0.0668
Dyspareunia (women)
0.341
0.1646
Sexual interest (women)
0.165
Score
TME
OP
Treatment
Group
iTNT (median follow-up: 21mo)
TNT (median follow-up: 48mo)
